# Management of COVID-19 ICU-survivors in primary care: - a narrative review

**DOI:** 10.1186/s12875-021-01464-2

**Published:** 2021-07-24

**Authors:** KFR Schmidt, J. Gensichen, S. Gehrke-Beck, R. P. Kosilek, F. Kühne, C Heintze, L. M. Baldwin, D. M. Needham

**Affiliations:** 1 Institute of General Practice and Family Medicine, Charité University Medicine Berlin, Berlin, Germany; 2 Institute of General Practice and Family Medicine, Jena University Hospital, Jena, Germany; 3 Institute of General Practice and Family Medicine, University Hospital of the Ludwig-Maximilians-University, Munich, Germany; 4 Department of Family Medicine, University of Washington School of Medicine, Seattle, WA USA; 5 Division of Pulmonary and Critical Care Medicine, Department of Physical Medicine and Rehabilitation, School of Medicine, Johns Hopkins University, Baltimore, MD USA

**Keywords:** Primary care, Post-intensive-care-syndrome, COVID-19

## Abstract

Many survivors of critical illness suffer from long-lasting physical, cognitive, and mental health sequelae. The number of affected patients is expected to markedly increase due to the COVID-19 pandemic. Many ICU survivors receive long-term care from a primary care physician. Hence, awareness and appropriate management of these sequelae is crucial. An interdisciplinary authorship team participated in a narrative literature review to identify key issues in managing COVID-19 ICU-survivors in primary care. The aim of this perspective paper is to synthesize important literature to understand and manage sequelae of critical illness due to COVID-19 in the primary care setting.

## Background

The COVID-19 pandemic is affecting primary care in many ways, including shortages of personal protective equipment, triage with limited resources, lack of therapeutic strategies, use of telemedicine, and economic constraints. However, another aspect of the pandemic is coming into view: recovery after treatment in an intensive care unit (ICU). Considerably more patients survive than die from the COVID-19, some after a long stay in an ICU. From more than two decades of research, we have substantial evidence that many ICU survivors do not return to their previous health status: Multiple physical, cognitive and mental health sequelae, known as the Post-Intensive Care Syndrome (PICS) [1], impact survivors’ return to work or meaningful activities for months or even years.

Similar to most chronically ill patients, the majority of ICU survivors continue to receive long-term aftercare from their primary care physicians. Within primary care, awareness of PICS may have been low: Until now, ICU survivors represent only a very small percentage of primary care patients. In addition, clinical signs associated with PICS are often similar to those caused by other chronic diseases. Furthermore, information flow between intensive care and primary care is impeded as these specialties represent the opposite ends of a spectrum within medical care. This existing situation may change with increasing numbers of COVID-19 survivors being discharged home and needing ongoing care. The Chartered Society of Physiotherapy even predicts “a tsunami of rehabilitation needs” [2] and also primary care physicians are likely to encounter substantially increased numbers of post-ICU COVID-19 patients. Consequently, the British Faculty of Intensive Care Medicine (FICM) realizes “a real opportunity to ensure full implementation of existing hospital and community based rehabilitation services for people recovering from critical illness.” [3].

The aim of this perspective paper is to synthesize important literature to support primary care providers in understanding and managing sequelae of critical illness due to COVID-19.

## Methods

We convened an interdisciplinary authorship team that has collaborated for up to 10 y on post-ICU aftercare research. In addition, several authors have been involved in guideline and review articles on post-ICU and post COVID-19 care [1, 4–6].

To prepare this perspective paper, the primary author identified and described key categories of challenges in post-ICU care of COVID-19 survivors. These categories were circulated to the authorship team for discussion and iterative refinement, until agreement was reached. Narrative or non-systematic literature reviews are employed to help support expert statements [7]. Thus, a narrative literature review was used to identify diagnostic and therapeutic strategies for each of these challenges. PubMed and Scholar databases were searched up to January 2021 on post-ICU and post COVID-19 care; the search strategy focused on systematic reviews, meta-analyses, national guidelines and randomized controlled trials, if available. In total, 63 relevant papers were identified. The resulting expert opinions are intended to support primary care for patients who experience sequelae of critical illness due to Covid-19.

## Results

### Post-ICU care

So far, evidence supporting structured ICU after-care is inconsistent: In randomized trials, outpatient post-ICU clinics have failed to demonstrate improved patient outcomes [8]. However, a primary care clinical assessment, within 90 days after hospitalization, is recommended by the UK’s NICE guideline [9], including reconciliation or elimination of inappropriate medications. To ensure optimal primary care assessment, effective information transfer and networks are needed. For example, detailed discharge notes from the hospital are essential, including data on respiration, mobility, swallowing, activities of daily life, as well as cognition and mental health status. Discharge letters handed out to the patient directly [10] provide a possible way to improve this transition between hospital-based and primary care.

Since recovery pathways and underlying diseases differ widely among ICU survivors, the reassessment process must be adapted individually. Table 1 provides major post-intensive care complications, including selected key symptoms, risk factors, screening instruments and treatment options. In summary, three key dimensions are recommended for primary care providers caring for post-ICU patients, inspired by the PICS concept: [1].

- Motor function, swallowing, and physical status.
- Mental health and cognitive function.
- Family and social health.

**Table 1:** Major post-intensive care complications

Sequelae	Description	Key symptoms	Example risk factors	Screening	Diagnosis	Treatment	Prognosis
Pulmonary function	• Obstruction, restriction and impaired diffusing capacity • Mostly following ARDS	• Shortness of breath • Reduced exercise capacity	Duration of mechanical ventilation	No consensus on recommended measure [6]	• Spirometry • Lung volumes • Diffusion capacity	Pulmonary rehabilitation program [5]	Generally: • mild impairment • improves during first year
Neuro-muscular function	• Joint contractures • Muscle weakness, including: -CIP -CIM -Disuse atrophy	• Reduced joint range of motion • Symmetric, distal and flaccid limb weakness • Reduced or absent deep tendon reflexes • Loss of peripheral sensation • Relative preservation of cranial nerve function	• Sepsis • Mechanical ventilation • Hyperglycemia • Use of glucocorticoids or neuromuscular blocking agents • Immobility/ bed rest	• Hand grip and/or Manual Muscle Test [11] • MRC scale [12]	• Nerve conduction study • Electromyography • Muscle ultrasound • Creatine kinase level (in ICU)	• Tailored rehabilitation across healthcare continuum, including PT and OT [5] • Home exercise [13] • Nutritional advice in case of malnutrition • Assistive devices	• CIP may recover more slowly than CIM • Abnormalities extend beyond five years • May not return to pre-ICU baseline status [5]
Physical function	Impairment in activities of daily living and walking distance		• Older age • Preexisting impairment	6MWT [14],4MGS [15] ADL/ IADL, (ICF) [16]			
Dysphagia	Swallowing impairment		• Prolonged intubation • Gastrointestinal comorbidity • ICUAW	Early consultation to a SLP [17]		Swallowing exercises with SLP [17]	Recoveries typically take more than 6 month
Mental Health	Depression	• Depressed mood • Loss of interest, fatigue	• Sedation • Traumatic/delusional memories of ICU • Pre-ICU psychiatric history • Female gender • Poverty • Not associated with severity of illness	**HADS** [18]* PHQ-2/9 [19]	DSM-5 diagnostic criteria, [20] semi-structured interview	• Psychotherapy • Antidepressants	May persist over first year [21]
	Anxiety	• Excessive worry, difficult to control		**HADS** [18]^*^ OASIS [22] GAD2/7 [23]		• CBT [5] • Anxiolytics	May have little improvement over first year [24]
	PTSD	• Intrusive memories • Avoidance of stimuli associated with the ICU • Dissociative reactions • Irritable behavior		**IES-6** [25]^*^ PTSS-10 [26]		• Talking about ICU experiences • Psychotherapy • Avoid benzodiazepines	• Onset may be delayed [27] • Little improvement in first year
Cognition	Impairments in • memory • attention • executive function • mental processing • visuo-spatial ability speed		• Prior cognitive deficit • Duration of ICU delirium • Older Age • Cerebral Hypoxia • Hypotension • Hypoglycemia	**MoCA** [28] MoCA Blind^*^	Exclusion of reversible causes for dementia as: • Hypothyroidism	• Cognitive rehabilitation • Assistance in organizing daily life	• May improve during first year • Residual deficits up to six years later
Family	PICS-F includes • Anxiety • Depression • PTSD • Complicated grief	see “Mental Health”	• Female gender • Younger age • Less education • Pre-ICU psychiatric history • Distance to hospital • Dissatisfaction with ICU communication	see “Mental Health”		• See “Mental Health” • Inclusion of family member into decision making • Involvement of trained nurse or social worker	PTSD and complicated grief may persist longer (over years) than depression and anxiety

Selected key symptoms, risk factors, screening instruments and treatment options of major post-intensive care complications. Modified from Mikkelsen et al. [29, 30], Desai et al. [31] and Schmidt et al. [32] without claim to completeness. Further scales are provided by Smith et al. 2020 [33].

* internationally agreed upon for acute respiratory failure survivors [6]

### Motor function, swallowing and physical status

ICU-Acquired Weakness (ICUAW), commonly caused either alone, or in combination, by muscle atrophy, Critical Illness Polyneuropathy (CIP) or Critical Illness Myopathy (CIM) has great impact on mobility and other activities of daily living [34]. Within primary care, early initiation of frequent physical therapy, occupational therapy, and nutrition advice may facilitate recovery from these conditions.

Around one third of patients with long-term mechanical ventilation have persisting dysphagia symptoms [17], increasing the risk for aspiration and pneumonia. Assessment by a speech and language therapist/ pathologist (SLP), including instrumental assessment of swallowing, may have occurred in the hospital setting prior to discharge. The need for ongoing speech and language therapy should be evaluated in the primary care setting.

In patients with Acute Respiratory Distress Syndrome (ARDS), which is common in severe cases of COVID-19 infections, long-lasting, clinically-important impairments in pulmonary function are surprisingly uncommon [35]. However, combined impairment in physical and cardiopulmonary status contribute to long-lasting reduction of exercise capacity (compared to a matched control group), as measured by the 6-Minute Walk Test [14]. (If the minimum 12 m-walkway is not available in the primary care practice, the 4-Meter Gait Speed Test may be considered.) [15] Early experiences among COVID-19 survivors suggest that early pulmonary rehabilitation, including breathing and movement training, may enhance recovery of respiratory and physical function [36].

After assessment of cardiorespiratory function by the primary care physician, breathing exercises and physical rehabilitation can be guided by physiotherapists, occupational therapists and/or primary care physician assistants, with expert input from physiatrists, as needed.

Beyond that, almost every organ system can be affected after intensive care, as listed for COVID-19 survivors in a Position Statement from the FICM [3]. Presenting all possible complications would go beyond the scope of this article. However, it is especially important to actively address potentially neglected topics, such as erectile dysfunction in male patients.

### Mental health and cognitive function

Many patients experience critical illness and ICU treatment as life-threatening events. New or worsened symptoms of depression, anxiety and/or post-traumatic stress disorder (PTSD) are common in the longterm. The etiology is complex - delirium, intrusive memories, use of sedative medications (e.g. benzodiazepines) and prior psychiatric history are commonly reported risk factors [21, 37]. Pandemic-related environmental factors, such as contact isolation, crisis mentality or overcrowded ICUs may heighten this risk [3, 38]. According to an observational study from Wuhan, almost all COVID-19 survivors showed symptoms of post-traumatic stress [39]. Psychiatrists expect upcoming rates of PTSD related to the pandemic similar to large scale disasters [40].

As many affected patients may avoid talking about these experiences, a proactive exploration of such symptoms, by the primary care physician, may be required [41], ideally supported by use of screening questionnaires [42], see Table 1. Talking about the ICU experience, and being listened to, are considered to be helpful - ideally using an ICU diary, if available [41]. Patients with severe or persistent symptoms may benefit from referral to a psychologist, psychiatrist or other mental health clinician. Among others, cognitive therapy has been highlighted recently to be applied in PTSD following critical illness [43].

Neurocognitive impairment among ICU survivors, associated with a history of delirium, hypoxia and/or hypotension in the ICU, can lead to significant impairment in daily life [44]. Common aspects of this impairment include reduced attention, memory and executive function. Reversible causes for cognitive impairment (e.g. hypothyroidism) should be excluded. Once this is done, the primary care physician may contribute to quality of life by assisting the patient and family in practically organizing daily life, along with specialized help from neuropsychologists and/ or cognitive rehabilitation therapy.

### Family and social health

Family members often experience the ICU course of their loved one closely. Thus, around 30% of them may suffer from relevant symptoms of anxiety, PTSD or depression during or after a critical illness of a relative [45]. Therefore, a separate term was introduced to raise awareness of these problems: PICS-Family [1]. Restricted access to inpatients in time of the pandemic may increase this particular risk [38]. Consequently, assessment of psychological symptoms should also be extended to a patient’s close family members [46]. Even if challenging due to time constraints, this may be especially necessary in the primary care setting.

Workplace reintegration is another important issue for consideration: Approximately 40% of critical illness survivors are unemployed at 12 months after discharge, while those who return to work might experience adverse changes to occupation or employment status [47]. Unemployment, in general, is associated with adverse mental health outcomes and might further aggravate the patients’ status. During the COVID-10 pandemic, it is unclear how the unprecedented economic shut-down may further exacerbate unemployment in ICU survivors.

Until now, there has been little evidence regarding specific interventions promoting return to work after critical illness. However, affected patients might benefit from multidisciplinary rehabilitation, including close coordination between their primary care physician, employer, and occupational medicine specialists [47].

### Options for support

ICU follow-up within primary care is challenging; additional support for patients and primary care providers is needed. Continuity of care in times of contact restrictions will be expanded increasingly to the virtual space. Patients may receive support by mobile applications promoting behavioural activation, breathing exercises or mindfulness [48]. Even a telephone-based intervention has been proven to increase coping skills following ICU discharge [49]. A growing selection of web resources supports diagnosis and treatment planning. For example, a recent “Practice Pointer” published in the BMJ provides general advice for management of post-acute COVID-19 patients in primary care [50]. Progress in a patient’s status can be tracked using a ‘functional reconciliation checklist’, which is considered to be useful, although its impact has not been evaluated [51]. Further resources are shown in Table 2.

**Table 2:** Selection of web resources on ICU follow up (adapted from Tingey et al. [52])

Organisation	Focus	Target group	Link
Johns Hopkins University Pulmonary & Critical Care Medicine, Baltimore, MD	Outcomes after Critical Illness and Surgery (OACIS)	Medical professionals	www.improveLTO.com https://www.hopkinsmedicine.org/pulmonary/research/outcomes-after-critical-illness/index.html
Wolters Kluwer, The Netherlands	Rehabilitation after Critical Illness		https://www.uptodate.com/contents/post-intensive-care-syndrome-pics#H457093
National Institute for Health and Care Excellence (NICE), UK			https://www.nice.org.uk/guidance/cg83
Intensive care patient support charity (ICUsteps), London, UK		Patients and Families	https://www.icusteps.org
Society of Critical Care Medicine (SCCM), Chicago, IL			https://www.sccm.org/MyICUCare/Home
Critical Illness, Brain Dysfunction, and Survivorship Center (CIBS), Nashville, TN	Delirium		https://www.icudelirium.org
ARDS Foundation, Dundee, IL	ARDS		https://ardsglobal.org/other-resources/

Standardized screening instruments possibly facilitate diagnostic assessment of impairments associated with PICS, as internationally agreed upon for acute respiratory failure survivors [6], see also Table 1. Patients with advanced age, preexisting chronic conditions, high intensity of intensive care and also ethnic minority background [29] are at highest risk for impairments; use of screening instruments should be focused on these groups. In addition, patients and their relatives can be referred to an intensive care support group and or follow-up clinic, if available. Furthermore, a detailed exercise instruction guide has been published to help COVID-19 survivors in physical rehabilitation at home [13].

However, primary care physicians need training in managing ICU survivors, as others have noted [42]. The authors advocate for integration of post-ICU care into primary care training and continuing medical education. Among other ideas, longitudinal clerkships to follow patient’s courses from ICU to primary care may provide a possible approach.

### Limitations

The information presented in this narrative review does not represent a clinical practice guideline, as it is limited by the non-systematic identification of studies as well as the missing formal evaluation of the risk of bias of the selected literature. Given the rapid development of research during the pandemic, new data may emerge and change any information presented herein. However, we consider the principle of multi-disciplinary collaboration will continue to be an important guiding principle in the field, with primary care physicians playing a key role in post-ICU management.

## Conclusion

Survivors of critical illness are at risk for long-lasting physical, cognitive and mental health sequelae. With the COVID-19 pandemic, these issues will grow in importance. Given the complexity and heterogeneity of the clinical course of ICU survivors, ICU follow-up requires multidisciplinary collaboration, which may be catalyzed by the COVID-19 pandemic. Primary care physicians play a key role in the management of post-ICU sequelae - due to their expertise in comprehensive medicine, coordination of care, embracing patients’ self-care and long-term knowledge of patients’ and their families’ medical history. The COVID-19 pandemic emphasizes the need for further research into post-ICU follow-up care, and its challenges in primary care.

## Data Availability

All: not applicable.

## Abbreviations

4MGS: 4 Meter Gait Speed Test [15]
6MWT: 6 Minute Walk Test [14]
ADL/ IADL: (Instrumental) Activities of daily living
ARDS: Acute Respiratory Distress Syndrome
CBT: Cognitive-Behavioral Therapy
CIM: Critical Illness Myopathy
CIP: Critical Illness Polyneuropathy
GAD-2/7: Generalized Anxiety Disorder 2/7-item [49]
HADS: Hospital Anxiety and Depression Scale [18]
ICF: World Health Organization’s International Classification of Functioning, Disability and Health Framework [16]
ICU: Intensive Care Unit
ICUAW: ICU-Acquired Weakness
IES-6: Impact of Event Scale- 6 item version [50]
MoCa (Blind): Montreal Cognitive Assessment (Blind) [24]
MRC: Medical Research Council [12]
OASIS: Overall Anxiety and Impairment Scale [21]
OT: Occupational Therapy
PHQ-2/9: Patient Health Questionnaire-2/9 [51]
PICS-F: Post-Intensive Care Syndrome-Family
PT: Physiotherapy
PTSD: *Post-Traumatic Stress Disorder*
PTSS-10: Posttraumatic Stress Scale-10 [52]
SIRS: Systemic Inflammatory Response Syndrome
SLP: Speech language pathologist
